# Rapid detection of illegal selective androgen receptor modulators in unregistered supplements using a combination of selected solid-state analytical methods

**DOI:** 10.5599/admet.2685

**Published:** 2025-06-08

**Authors:** Izabela Jendrzejewska, Lubos Cehlarik, Tomasz Goryczka, Ewa Pietrasik, Natalia Pawlik, Josef Jampilek

**Affiliations:** 1 Institute of Chemistry, University of Silesia, Bankowa 12, 40-007 Katowice, Poland; 2 Department of Criminalistics and Forensic Science, Academy of the Police Force in Bratislava, Sklabinska 8414/1, 835 17 Bratislava, Slovakia; 3 Institute of Materials Science, University of Silesia, Bankowa 12, 40-007 Katowice, Poland; 4 Department of Analytical Chemistry, Faculty of Natural Sciences, Comenius University, Ilkovicova 6, 842 15 Bratislava, Slovakia

**Keywords:** Pharmaceutical crime, unregistered dietary supplements, counterfeits, Raman spectrometry, differential scanning calorimetry, X-ray powder diffraction

## Abstract

**Background and purpose:**

Pharmaceutical crime is becoming an increasingly serious threat. For customs officers and police officers, minimal or no sample preparation before analysis is essential when detecting prohibited compounds/counterfeit products, ideally using on-site analysis.

**Experimental approach:**

Unregistered dietary supplements containing anabolic substances, specifically selective androgen receptor modulators (SARMs) such as andarine, ligandrol, ostarine, and testolone became the subject of investigation. Dietary supplements with these SARMs are often found illegally in various fitness centres and can be purchased online. Furthermore, these illegal supplements may not contain the declared SARMs at all or may contain incorrect amounts. Analytical methods such as Raman spectroscopy, differential scanning calorimetry, thermogravimetry, and X-ray powder diffraction were chosen to identify these banned SARMs in the illegal products.

**Key results:**

Using a combination of these fast and direct analytical techniques, in particular, Raman spectroscopy, it was found that out of 16 samples, SARMs were confirmed in 9 samples, while no testolone (4 samples), ostarine (2 samples), or andarine (1 sample) was reliably identified in 7 samples.

**Conclusion:**

Overall, almost half of the analyzed samples of unregistered/illegal sports dietary supplements purchased anonymously online in the Slovak Republic with declared content of at least one SARM did not contain what is declared on the label. Thus the combination of several solid-state analytical techniques without complex sample preparation has proven valuable for rapid identification of APIs in supplements.

## Introduction

Pharmaceutical crime is becoming an increasingly severe threat [1,2]. Counterfeiting of pharmaceutical products, packaging, and documentation is directly related to theft, fraud, abuse, smuggling, illegal trading, and legalisation of the proceeds of crime [3,4]. Illegal markets for counterfeit drugs, pharmaceuticals, and dietary supplements are attractive to counterfeiters mainly due to high margins, low risk of detection/prosecution, and low penalties [5-10].

One of the possible definitions of a dietary supplement says that it is a product intended to supplement the diet that bears or contains one or more dietary ingredients. A dietary supplement must be labelled as intended for ingestion and not be represented as a conventional food or a sole item of a meal or diet [11-13]. Athletes consume dietary supplements to increase muscle energy reserves, increase muscle mass, reduce body fat, and provide overall body stimulation during sports activities and subsequent regeneration [14]. Dietary supplements are readily available through (illegal) athlete-focused online stores, so there must be an emphasis on educating coaches, athletes, and the public [15,16]. It is confirmed that several sport dietary supplements can be intentionally/unintentionally contaminated with prohibited substances [17-19]. In some cases, dietary supplements do not even contain the declared active pharmaceutical ingredients (APIs) and their quantities; on the contrary, they contain substances and amounts not listed on the package labels. In contrast, the purity of chemical substances is not guaranteed [20]. The presence of illegal substances in dietary supplements can cause positive doping findings, but at the same time, it can negatively affect the health of athletes [21].

Selective androgen receptor modulators (SARMs) have been known since the late 20th century as androgen receptor agonists/partial agonists [22]. They were developed to eliminate the side effects of androgenic steroids to be used as supportive therapy in the treatment of cancer, HIV, and various chronic and/or degenerative diseases [23]. It is a highly heterogeneous class of compounds with both steroidal and non-steroidal structures [24,25]. Although no SARMs are currently approved for therapeutic use [26,27], they have become the subject of black-market trade in the form of unregistered dietary supplements, which are abused primarily by athletes for performance enhancement due to their anabolic properties and ability to stimulate androgen receptors in the bones and muscles [28]. The US FDA and the US Anti-Doping Agency have warned consumers that "body-building" dietary supplements containing SARMs are dangerous and illegal, and their use poses an immediate health risk [29-34]. SARMs pose the risk of acute liver and kidney damage, shrinking of the testicles, breast enlargement, infertility, masculinization of women, heart attack, and stroke, can cause short stature in children, increase the risk of drug and alcohol abuse, and can disrupt the regulation of blood levels lipids. At the same time, the long-term effects on the human organism are unknown [35-38].

Thus, SARMs are chemical substances banned in official sports worldwide and listed in section S1.2 “Other anabolic agents” of the Prohibited List of the World Anti-Doping Agency (WADA) [39,40]. In 2011, the first adverse analytical finding of andarine in the context of sports activities was published [41]. Since then, WADA has recorded other cases of abuse of SARMs, especially the already mentioned andarine, as well as ostarine, ligandrol, and testolone [42-49]. SARMs are also starting to be abused in cases promoting farm animal growth [50,51].

Unregistered dietary supplements with declared and presumed SARM content, sold on the black market, do not meet the necessary standards of quality, efficacy, and safety, which poses a risk to users' health [52,53]. Therefore, continuous market monitoring [54-57] to detect the presence of these substances is proposed [58-61]. It primarily identifies and quantifies SARMs in dietary supplements to verify their declared amount on the labels [62]. The discovery of such pharmaceutical products is a very complex process regarding the exercise of expertise and the expenditure of funds. The challenge for all countries of the world is e-shops and internet pharmacies that sell these products cheaply, while people's willingness to risk buying from unverified sources is increasing, which can lead to severe damage to health [63-66].

**Figure 1.**
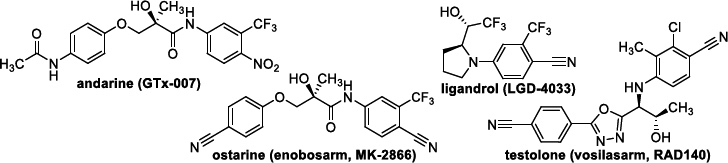
Structures of investigated compounds.

Unregistered dietary supplements containing SARMs, such as andarine, ligandol, ostarine and testolone (Figure 1), became the subject of investigation. Supplements with these SARMs are often found illegally in various fitness centres and can be bought illegally online. For customs officers and police officers, minimal or no sample preparation before analysis is essential when detecting prohibited compounds/counterfeit products, ideally using on-site analysis. Therefore, direct analytical methods, especially solid-state techniques, allowing easy and fast identification/detection of prohibited APIs based on compliance with the standard, are becoming increasingly important. To identify these four prohibited SARMs in 16 illegal products (see section Materials) purchased online in e-shops, Raman spectroscopy (RS), differential scanning calorimetry (DSC), thermogravimetry (TG), and X-ray powder diffraction (XRD) were chosen, which allow immediate analysis of samples without complex preparation. In particular, RS has become an essential tool for customs and police officers to directly detect prohibited compounds in situ [67-70] in contrast to, *e.g.* laborious, time-consuming, expensive, and laboratory-bound HPLC-MS. This article builds upon previous publications that evaluate pharmaceutical preparations using selected solid-state analytical methods by employing similar methodologies [71-74].

## Experimental

### Materials

As standards, the following APIs have been used: andarine (Sigma Aldrich-Merck, St. Louis, MO, USA), ostarine (Santa Cruz Biotechnology, Inc., Heidelberg, Germany), ligandrol (Toronto Research Chemicals Inc., Toronto, ON, Canada), and testolone (BioVision Inc., Milpitas, CA, USA).

Sixteen unregistered dietary supplements for athletes declaring the content of at least one of the SARMs (andarine, ostarine, ligandrol, or testolone) were analysed by solid-state analytical methods with no sample preparation. All unregistered dietary supplements were purchased anonymously online in the Slovak Republic (in the Slovak online environment) through online shops with a declared company headquarters in Slovakia and delivered by courier. The names, composition and content of SARMs are listed in Table 1; it should be noted that these are the data provided on the labels of individual products by the manufacturers.

**Table 1.** Summary of information about investigated products, unregistered dietary supplements

No.	Product name*	Manufacturer (country of origin)*	1 capsule weight, mg	Declared API content in 1 capsule*, mg	Excipients (other components)*	API in 1 capsule, wt.%
Andarine (GTx-007)						
1	Patriot Andarine^®^	Underground Labs (n.s.)	703	15	n.s.	2.1
2	Gelabs Andarine^®^	Gelabs (USA)	920	25	*N*-acetyl-L-cysteine, vitamin E	2.7
3	Cartel Labz Andarine S-4^®^	Cartel Labz (USA)	276	30	Gelatin, magnesium stearate	10.9
4	Magnus Pharmaceuticals Andarine S-4^®^	Magnus Pharmaceuticals (n.s.)	457	25	Rice flour, organic rice, gelatin, magnesium stearate	5.5
Ligandrol (LGD-4033)						
5	Red Army Bolshevik Blood^®^	Red Army Labs (n.s.)	593	2	Rice flour, gelatin	0.3
6	Swiss Pharmaceuticals Ligandrol^®^	Swiss Pharmaceuticals (Swiss)	448	10	Gelatin, magnesium stearate	2.2
7	Fusion Supplements LGD^®^	Fusion Supplements (n.s.)	244	10	Corn starch, gelatin	4.1
8	iMuscle Ligandrol LGD^®^	iMuscle European Division (n.s.)	427	8	n.s.	1.9
Ostarine (MK-2866)						
9	Dark Labs Ostarine MK-2866^®^	Dark Labs (n.s.)	436	10	Rice flour, gelatin, magnesium stearate, FD&C Blue#1, FD&C Red#40	2.3
10	Savage Line Labs MYO-STA Ostarine MK-2866^®^	Savage Line Labs (USA)	416	10	Rice flour, gelatin, magnesium stearate	2.4
11	Red Army Cold War^®^	Red Army Labs (n.s.)	603	10	Rice flour, gelatin	1.7
12	Swole Labs MK-2866 Ostarine^®^	Swiss Labs (Swiss)	690	20	Rice flour, gelatin, magnesium stearate	2.9
Testolone (RAD140)						
13	Biogenic Pharma Testolone (RAD-140)^®^	Biogenic Pharma (Canada)	281	5	Gelatin, magnesium stearate	1.9
14	Lawless Labs Testolone Sarm RAD-140^®^	Lawless Labs (USA)	741	10	Rice flour, gelatin, calcium silicate	1.4
15	German Pharma R (RAD-140)^®^	German Pharma (UK)	380	5	Rice flour, gelatin, magnesium stearate, stearic acid, silica	1.3
16	Bio Molecule RAD 140 Testolone^®^	Bio molecule LLC (USA)	306	10	Rice flour, organic rice, gelatin, magnesium stearate	3.3

*information taken from the labels of the investigated products; n.s. = not specified on the product

### Methods

The samples were ground perfectly in an agate mortar for adequate analyses to obtain a homogeneous powder. In the next step, such prepared samples were tested using a Philips X-Ray PW3050 X'Pert Diffractometer (Malvern Panalytical, Malvern, UK). Thermal analysis was done using a Labsys Evo apparatus (Setaram Inc., Cranbury, NJ, USA). The technical details of these methods are described in ref. [72,74]. Raman spectroscopy was performed using a Raman DXRxi spectrometer (Thermo Fisher Scientific, Waltham, MA, USA); for technical details, see Table 2.

**Table 2.** Configuration of the Raman spectrometer during experiments

Wavelength of laser	780 nm
Power of laser	24 mV
Time of exposition	1 s
Number of scans	100
Confocal microscope lens	10×, 50×

For these investigations, easy, reliable, and inexpensive methods were chosen. As a non-destructive method, X-ray powder diffraction (XRPD) is suitable for pharmaceutical control. This method can easily distinguish counterfeit samples from original ones. Diffraction patterns can serve as fingerprints of investigated samples. The intensity of diffraction lines and their characteristic 2 *θ* values can be used in qualitative phase analysis, in which powder diffraction databases are applied. Thermal analysis, including differential scanning calorimetry (DSC) and thermogravimetry (TG), is used to determine the composition and properties of new substances and the compatibility of the tested substances with the reference ones. The Raman technique is a sensitive, non-destructive, selective method that can be used *in situ*. No sample preparation is required. Thanks to microelectronics and computer science development, the Raman technique is becoming an increasingly widely used research tool. These techniques are not time-consuming and can be used even by employees who are not highly experienced [75].

Due to the lack of standards, DSC/TG analyses were impossible for pure APIs. For the interpretation of the DSC/TG results of the investigated APIs, the accessible data were used. It is known that andarine melts at 70 to 74 °C [76], ostarine at 71 to 80 °C [77,78], ligandrol at 105 to 106 °C [79], and testolone at 170-171 °C [80]. However, in Ref. [81], the melting points for andarine and ostarine differ: 138 °C for ostarine and 156 °C for andarine.

## Results and discussion

### Study of products containing andarine

**Figure 2.**
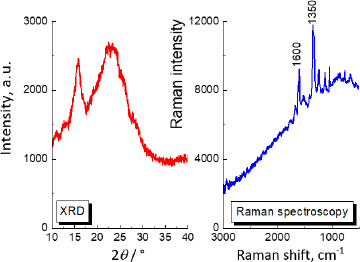
XRD and Raman spectra for pure andarine

Firstly, the XRD and Raman analyses for pure andarine were done to compare them with further investigation samples. The results are depicted in Figure 2.

Generally, the most crucial criterion in the XRD qualitative analysis, confirming the presence of a given phase, is the agreement between the positions of diffraction lines in the recorded diffraction image and those in the reference image. The difference should not exceed 0.2° [82]. For andarine, the strongest diffraction lines were recorded at the following 2 *θ* angles: 15.667°, 22.517°. The comparison of diffraction data for pure andarine API and the investigated products is shown in Table 3, where *J/J*_max_ × 100 is a relative intensity of diffraction lines (ratio of the intensity of the chosen diffraction line to the diffraction lines with maximum intensity, multiplied by 100), and *d*_hkl_ is an interplanar distance.

**Table 3.** Comparison of diffraction data for andarine samples with the XRD data for pure andarine (pattern)

No. of diffraction line	2*θ / °*		*J*/*J*_max_ × 100 / %		|Δ2 *θ| / °*	*d*_hkl_ / nm	
	Pattern	Sample	Pattern	Sample		Pattern	Sample
Patriot Andarine^®^							
1.	22.517	22.652	100	88	0.135	0.3945	0.3992
Gelabs Andarine^®^							
Lack of visible proper diffraction lines							
Cartel Labs Andarine^®^							
1.	22.517	22.395	100	100	0.122	0.3945	0.3967
Magnus Pharmaceuticals Andarine^®^							
1.	22.517	22.597	100	75	0.080	0.3945	0.3932

**Figure 3.**
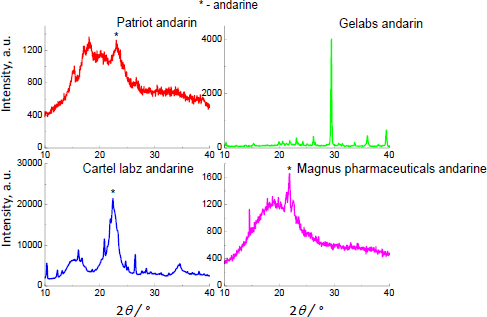
XRD images for products containing andarine

The XRD images of three products, Patriot Andarine^®^, Cartel Labs Andarine^®^ and Magnus Pharmaceuticals Andarine^®^, correspond to the XRD image of pure andarine (see Figure 3). The position of the strongest diffraction line is in good agreement with the position of the diffraction line of pure andarine (Table 3). The values of |Δ2*θ*| are smaller than 0.2°. For Patriot Andarine^®^, Cartel Labs Andarine^®^, and Magnus Pharmaceuticals Andarine^®^, a high background is observed, similar to the XRD images of pure andarine. It confirms the presence of amorphous substances in the phase composition of investigated products. Only for Gelabs Andarine^®^, the XRD image is quite different from the XRD images of the rest of the supplements. The diffraction pattern is typical for crystalline ingredients creating supplement Gelabs Andarine^®^. The lack of proper diffraction lines is observed. The preferred orientation (texture) can be met in Gelabs Andarine^®^. It is indicated by a strong diffraction line at a 2 *θ* value of about 29° [81]. In the presence of texture, the diffraction lines from andarine can get lost in the background, especially the share of andarine. However, the amount of andarine is above the limit detection – 2.71 wt.% (see Table 1). The X-ray detection limit ranges from 0.1 to 1 wt.% per phase, while the limit of detection (LOD) is assumed to be approx. 1 % [83].

**Figure 4.**
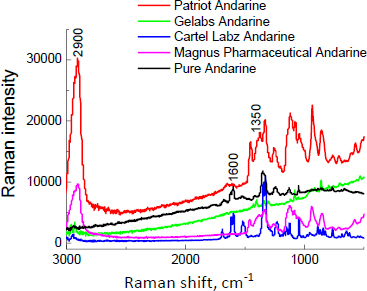
Raman spectra of products with andarine

The Raman spectrum for andarine, visible in Figure 2, shows two strong lines at Raman shift equal to 1600 and 1350 cm^-1.^ The Raman spectra of supplements containing andarine are illustrated in Figure 4. The Raman spectrum for pure andarine is presented in this figure (black line) for comparison. As is evident, the Raman intensity of Gelabs Andarine^®^ is much lower than that of other products, although the amount of API should be comparable to Patriot Andarine^®^ (2.1 wt.%, see Table 1). The relatively low Raman signals from andarine in Gelabs Andarine^®^ could be influenced by the sample’s fluorescence.

**Figure 5.**
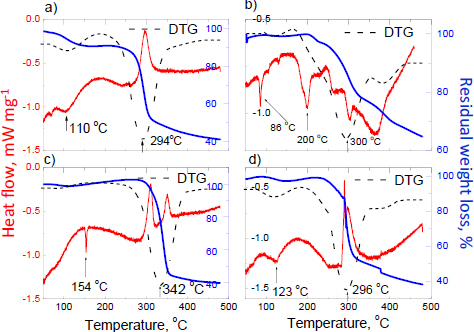
DSC, TG, and DTG curves for products containing andarine: a) Patriot Andarine^®^, b) Gelabs Andarine^®^, c) Cartel Labs Andarine^®^ and d) Magnus Pharmaceuticals Andarine^®^

Thermal analysis of the investigated products with andarine is presented in Figure 5, where the shapes of the DSC, TG, and DTG curves for Patriot Andarine^®^, Cartel Labs Andarine^®^, and Magnus Pharmaceuticals Andarine^®^ are similar. A. Turza *et al.* [81] state that andarine melts at 156 °C. The best agreement is observed for Cartel Labs Andarine^®^. An endothermic peak originates from andarine, is observed at 154 °C, and indicates the melting process without mass loss. According to the manufacturer's declaration on the label, the amount of andarine in this supplement is the highest (10.9 wt.%, see Table 1). The endothermic peak at 342 °C is assigned to the decomposition and degradation process.

For smaller amounts of andarine (the amount expected according to the information on the labels), an endothermic peak typical of andarine is invisible. For Patriot Andarine^®^ and Magnus Pharmaceuticals Andarine^®^, the first endothermic peaks at 110 and 123 °C, respectively, point to the start of the melting process. Patriot Andarine^®^, Gelabs Andarine^®^, and Magnus Pharmaceuticals Andarine^®^ decompose at about 300 °C. The endothermic peaks visible for Gelabs Andarine^®^ indicate that the melting process occurs in several steps. For Patriot Andarine^®^, Cartel Labs Andarine^®^, and Magnus Pharmaceuticals Andarine^®^, exothermic peaks are observed on DSC curves. It confirms the presence of amorphous substances included in the tested supplements.

For Gelabs Andarine^®^ (2.7 wt.%, see Table 1), the shape of the DSC/TG and DTG curves is dissimilar to the results of thermal measurements for the rest of the products. Several endothermic peaks indicate several melting processes of substances included in the supplements. This phenomenon points out the alternative phase composition of this product (more crystalline additional substances). The mass loss is about 60 % for products containing andarine, except Gelabs Andarine^®^, for which the mass loss is about 35 %. It confirms the more significant contribution of crystalline phases to this product.

The results of the XRD study, Raman spectroscopy, and thermal analysis obtained for supplements with andarine agree. That means that the results complement and confirm each other, the presence (or not) of the API in examined samples. The presence of API declared by the manufacturer in Gelabs Andarine^®^ seems questionable.

### Study of products containing ligandrol

**Figure 6.**
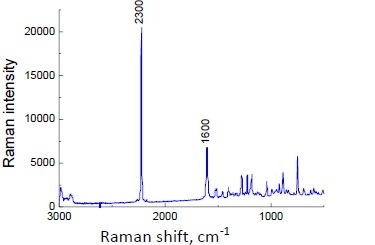
Raman spectrum for ligandrol

**Figure 7.**
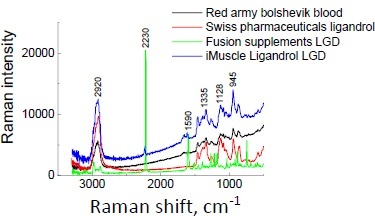
Raman spectra for supplements containing ligandrol

The Raman spectra of pure ligandrol and supplements containing ligandrol are illustrated in Figures 6 and 7, respectively. For ligandrol, a strong Raman shift is visible at 2230 cm^-1^, typical for the –C≡N moiety. The results of Raman spectroscopy indicate that Raman shifts at 2230 cm^-1^ are present for Fusion Supplements LGD^®^ and iMuscle Ligandrol LGD^®^. In Red Army Bolshevik Blood^®^, the amount of ligandrol is the lowest according to the information on the product label (0.3 wt.%, see Table 1), while in Swiss Pharmaceuticals Ligandrol^®^ (2.2 wt.%, see Table 1), the quantity of pure ligandrol is comparable to that in iMuscle Ligandrol LGD^®^ (1.9 wt.%) according to the information on the product labels. Lack of confirmation may indicate a smaller amount of ligandrol, below the detection limit, or difficult detection due to the coexisting phases.

**Figure 8.**
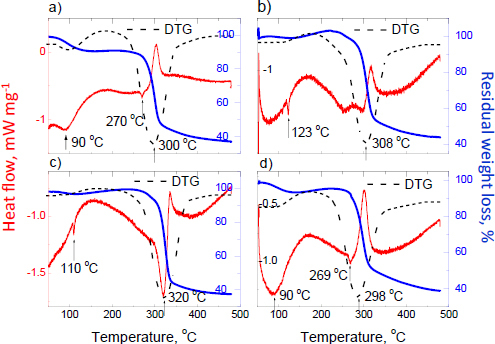
DSC/TG/DTG curves for products containing ligandrol: a) Red Army Bolshevik Blood^®^, b) Swiss Pharmaceuticals Ligandrol^®^, c) Fusion Supplements LGD^®^ and d) iMuscle Ligandrol LGD^®^

The DSC/TG/DTG curves for products with ligandrol are demonstrated in Figure 8. The shape of the DSC/TG curves for all investigated supplements containing ligandrol is similar. The API structure is destroyed at a temperature of about 300 °C. The exothermic peak on the DSC curve is about 300 °C, which can indicate the presence of rice flour [84].

The endothermic peaks, visible for Red Army Bolshevik Blood^®^ and iMuscle Ligandrol LGD^®^ at about 270 °C, corroborate the higher amount of this component in these two supplements compared to the other products. However, the broadened peak at 90 °C shows that the melting process starts at this temperature for these two samples. The endothermic peak, which indicates ligandrol, is detected for Fusion Supplements LGD^®^. It is corroborated by the sharp endothermic peak at 110 °C (pure ligandrol melts at 105-110 °C). It is possible because, according to Table 1 and the label information, the expected amount of ligandrol in Fusion Supplements LGD^®^ is the highest among all supplements of this group (4.1 wt.%, Table 1). For Swiss Pharmaceuticals Ligandrol^®^, the endothermic peak at 123 °C marks the melting process of any ingredient of this product. The visible exothermic peaks on DSC curves confirm the presence of amorphous compounds in the composition of the supplements with ligandrol. The mass loss is about 60 % for all products containing ligandrol.

### Study of products containing ostarine

**Figure 9.**
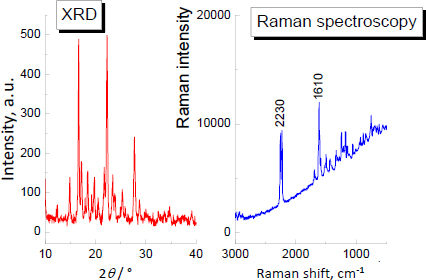
XRD and Raman spectra for pure ostarine

The XRD study and Raman spectroscopy results of pure ostarine are shown in Figure 9.

**Figure 10.**
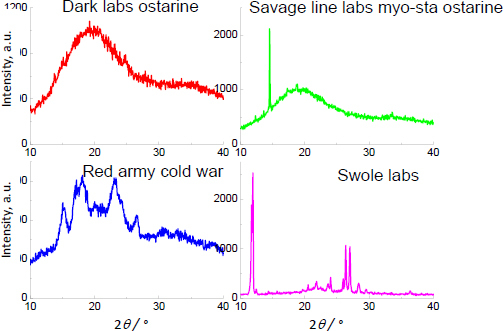
XRD images of supplements containing ostarine.

For ostarine, the strongest diffraction lines were recorded at the following 2 *θ* angles: 16.529, 22.264 and 27.724°. The XRD studies for supplements containing ostarine are visible in Figure 10. No proper diffraction lines originating from ostarine have been detected in all XRD images.

The contribution of ostarine in the investigated samples is in the range of 1.66 to 2.90 wt.% (Table 1). The amount of active substance is probably below the detection limit, or the weak ostarine diffraction lines may be hidden in a high background. On the other hand, on the diffraction pattern of Savage Line Labs MYO-STA Ostarine MK-2866^®^ and Swole Labs MK-2866 Ostarine^®^, strong diffraction lines are observed at 14.55 and 11.90°, respectively. This phenomenon can indicate the texture of the samples. For Dark Labs Ostarine MK-2866^®^, Savage Line Labs MYO-STA Ostarine MK-2866^®^ and Red Army Cold War^®^, the high background and broadened diffraction lines evidence the amorphous character of the examined supplements. Swole Labs MK-2866 Ostarine^®^ exhibits the characteristics of a crystalline API, but the visible diffraction lines do not correspond to the pattern of the XRD image of ostarine.

**Figure 11.**
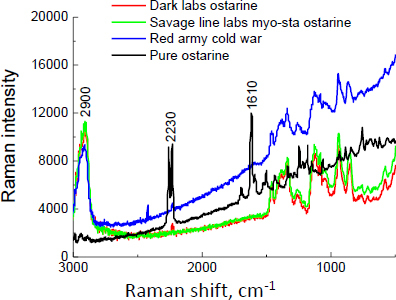
Raman spectra for supplements containing ostarine

The Raman spectra for supplements containing ostarine are shown in Figure 11, with the Raman shift at 2230 cm^-1^ (characteristic for the –C≡N moiety) visible for Dark Labs Ostarine MK-2866^®^ and Red Army Cold War^®^, confirming the presence of ostarine in the tested products. For Swole Labs MK-2866 Ostarine^®^, the Raman spectrum was impossible to obtain due to the strong fluorescence. Thus, although the manufacturers declare on the labels for Savage Line Labs MYO-STA Ostarine MK-2866^®^ and for Swole Labs MK-2866 Ostarine^®^ the content of 10 and 20 mg API in 1 capsule, respectively (*i.e.* 2.4 and 2.9 wt.%, respectively), no characteristic Raman band was found, which may indicate a lower amount of ostarine in these products than declared by the manufacturers.

**Figure 12.**
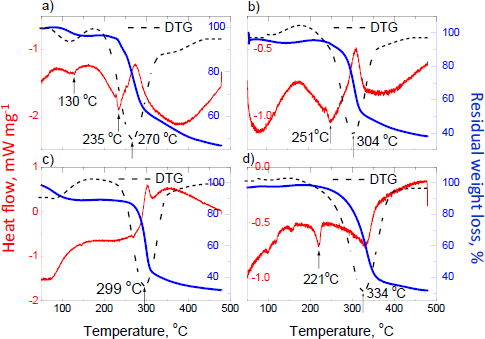
DSC, TG, and DTG curves of products containing ostarine: a) Dark Labs Ostarine MK-2866^®^, b) Savage Line Labs MYO-STA Ostarine MK-2866^®^, c) Red Army Cold War^®^ and d) Swole Labs MK-2866 Ostarine^®^

The results of the DSC/TG analysis are presented in Figure 12. The DTG curves show that the decomposition and degradation of samples are observed in the temperature range between 270 °C and 334 °C. According to the publicly available data, the melting point of ostarine is 132 to 138 °C [81]. In the temperature range between 50 and 270 °C, only the DSC curve of Dark Labs Ostarine MK-2866^^®^^ shows two endothermic peaks. The first weak peak (at 130 °C) indicates the small amount of ostarine in this product (according to the manufacturer's declaration, see Table 1, it should be 2.3 wt.%), while the second endothermic peak can indicate the rice flour's melting point, which is 260 to 280 °C [84]. For the rest of the investigated supplements, the first endothermic peak, indicating ostarine, is absent due to the lower content of ostarine in these products; the detected endothermic peaks are associated with the presence of additional substances in the phase composition of these supplements (Table 1). The shape of the DSC curves of Dark Labs Ostarine MK-2866^®^, Savage Line Labs MYO-STA Ostarine MK-2866^®^, and Red Army Cold War^®^ is similar, while the DSC curve for Swole Labs MK-2866 Ostarine^®^ is different. Dark Labs Ostarine MK-2866^®^, Savage Line Labs MYO-STA Ostarine MK-2866^®^, and Red Army Cold War^®^ have amorphous characters noticeable in XRD images. Swole Labs MK-2866 Ostarine^®^ has a more crystalline character of phase composition, corroborated by the DSC curve shape. The mass loss is about 60% for all products containing ostarine.

### Study of products containing testolone

**Figure 13.**
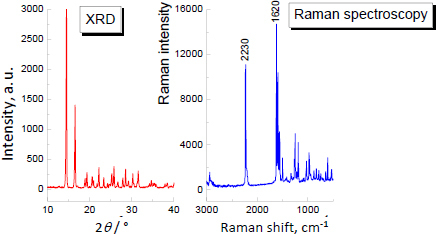
XRD and Raman spectra for pure testolone

The XRD study and Raman spectroscopy results of pure testolone are depicted in Figure 13.

**Figure 14.**
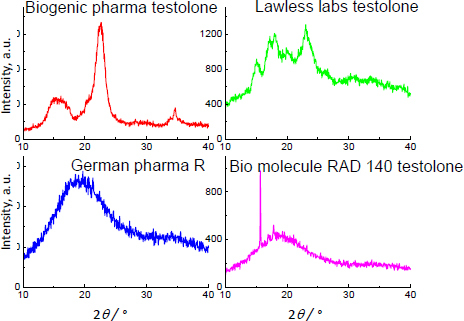
XRD images of products containing testolone.

For testolone, the strongest diffraction lines were recorded at the following 2 *θ* angles: 14.448 and 16.449°. The results of the XRD study for supplements containing testolone are presented in Figure 14.

The diffraction lines, originating from testolone, have not been detected. The testolone content in the investigated samples is 1.78-3.27 wt.% (Table 1). Theoretically, the diffraction lines should be recorded. The shape of XRD images revealed the amorphous state of the analyzed samples. A low testolone amount can cause a lack of proper diffraction lines, while the testolone content is above 1 wt.% (Table 1). The intensity of testolone diffraction lines is minimal; thus, the diffraction lines can be hidden in a high background. One strong diffraction line is observed for Bio Molecule RAD 140 Testolone^®^. It can illustrate the presence of crystalline substances in this drug's phase composition or texture.

**Figure 15.**
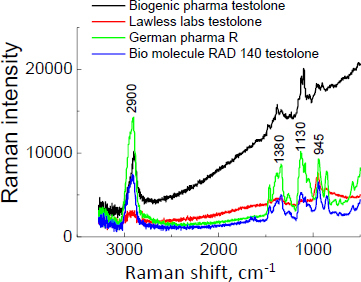
Raman spectra for products containing testolone.

The Raman spectra for supplements with testolone are presented in Figure 15. The characteristic Raman shift (2230 cm^-1^), confirming the presence of testolone, is not detected. The amount of the testolone API in the studied products is likely smaller than what was declared by producers or is too low to detect. Moreover, the lack of a typical Raman shift (at 2230 cm^-1^) is caused by difficulty in detection due to the coexisting phases.

**Figure 16.**
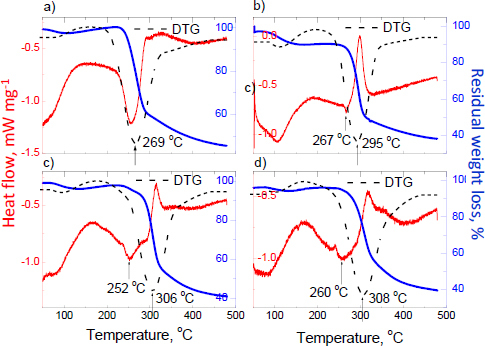
DSC, TG, and DTG curves for products containing testolone: a) Biogenic Pharma Testolone (RAD-140)^®^, b) Lawless Labs Testolone Sarm RAD-140^®^, c) German Pharma R (RAD-140)^®^ and d) Bio Molecule RAD 140 Testolone^®^

The DSC/TG/DTG curves of products containing testolone are depicted in Figure 16.

Thermal analysis of supplements with testolone revealed that this group of products melts in the range of 269 to 308 °C. The detected endothermic peak at 252 to 267 °C is observed for all products. This peak points out the presence of rice flour in the composition of these products [84]. The shape of the DSC curves and visible exothermic peaks confirm the presence of amorphous ingredients in the phase composition of the tested supplements. The lack of a typical endothermic peak confirming the presence of ligandrol is caused by a small amount of ligandrol, which agrees with the XRD results. The mass loss is about 60 % for all products with testolone.

## Conclusions

Based on the performed measurements of the unregistered dietary supplements with declared SARM content by the manufacturer, it was found that most of the investigated products are in an amorphous state. Exceptions are Gelabs Andarine^®^ and Swole Labs MK-2866 Ostarine^®^. The mass loss is about 60% for the examined products, except Gelabs Andarine^®^, for which the mass loss is about 35%. It confirms the more significant contribution of crystalline phases in most products. The thermal analysis allowed us to determine the thermal characteristics of all the investigated APIs/supplements. Thermal measurements confirmed the XRD study; for most APIs in the individual supplements, the shape of the DSC curve shows, at about 300 °C, the exothermic peak characteristic for rice flour, which is present in most products. This excipient is confirmed by Raman shift at 2900 cm^-1^, visible on Raman spectra. For ostarine, ligandrol, and testolone, a strong Raman shift is visible at 2230 cm^-1^, typical for the –C≡N moiety. It allows us to distinguish andarine from other molecules. Raman spectroscopy revealed the Raman spectra for the SARMs in the dietary supplements. The characteristic Raman shift at 2230 cm^-1^, typical for the -C≡N moiety, is visible in some supplements with ligandrol and ostarine. For supplements with testolone, this Raman shift is absent. Lack of this characteristic Raman shift can indicate a lower amount of API than declared. Also, the Raman intensity of one supplement containing andarine is very low. Overall, out of 16 purchased samples available online, SARMs were confirmed in 9 samples, while no testolone (4 samples), ostarine (2 samples), or andarine (1 sample) was reliably identified in 7 samples.

Studies combining several techniques, such as thermal analysis, X-ray study, and Raman spectroscopy, can help focus attention on these groups of supplements and guide the rapid identification of APIs in these supplements. The applied research methods are simple and fast, require small samples, identify APIs, and detect any changes in the phase composition of these products. On the other hand, an X-ray powder diffractometer is not standard equipment in routine laboratories. However, this is not the case for a DSC instrument and a Raman spectrometer, which are commonly present. This approach should be understood as the first step in the fast forensic analysis of products suspected of containing illegal SARMs with no sample preparation required.
